# Molecular Mechanisms of Eosinophilic Esophagitis

**DOI:** 10.3390/ijms222413183

**Published:** 2021-12-07

**Authors:** Yury V. Zhernov, Sonya O. Vysochanskaya, Vitaly A. Sukhov, Olga K. Zaostrovtseva, Denis S. Gorshenin, Ekaterina A. Sidorova, Oleg V. Mitrokhin

**Affiliations:** 1 Department of General Hygiene, F. Erismann Institute of Public Health, I.M. Sechenov First Moscow State Medical University (Sechenov University), 119435 Moscow, Russia; m.olochnik@yandex.ru (S.O.V.); sukhov_v_a@staff.sechenov.ru (V.A.S.); ernilasengal1@gmail.com (O.K.Z.); dgorhenin63@gmail.com (D.S.G.); Ekaterina_sidorovaa@mail.ru (E.A.S.); mitrokhin_o_v@staff.sechenov.ru (O.V.M.); 2 Department of Chemistry, Lomonosov Moscow State University, 119991 Moscow, Russia

**Keywords:** food hypersensitivity, eosinophilic esophagitis, non-IgE-mediated food allergy, pseudo-allergic reactions

## Abstract

Food hypersensitivity is a group of diseases arising from a specific immune response that reproduces on exposure to a given food. The current understanding of molecular mechanisms and immunopathology of non-IgE-mediated/mixed food hypersensitivity, e.g., eosinophilic esophagitis, contains many gaps in knowledge. This review aims to provide a modern classification and identify the primary diseases of non-IgE-mediated/mixed food hypersensitivity reactions, delineate the distinctive molecular features, and discuss recent findings in the immunopathology of eosinophilic esophagitis that may become a basis to develop valid biomarkers and novel therapies for this disease. Eosinophilic esophagitis is a recently recognized allergic-mediated disease with eosinophil-predominant esophagus inflammation. Its pathogenesis is a complicated network of interactions and signaling between epithelial, mesenchymal, and immune cells on molecular and intercellular levels. Alterations produced by overactivation of some cytokine signaling pathways, e.g., IL-13 or thymic stromal lymphopoietin (TSLP), were evolved and observed in this review from the viewpoints of molecular, genetic, epigenetic, and transcriptomic changes. Despite substantial experimental data, the reliable and representative mechanism of eosinophilic esophagitis pathogenesis has yet to show itself. So, the place of esophagitis between mixed and non-IgE-mediated allergic disorders and between eosinophilic gastrointestinal disorders currently seems vague and unclear.

## 1. Introduction

Food hypersensitivity (FH) is a group of diseases arising from a specific immune response that reproduces on exposure to a given food. FH can be broadly classified into IgE-mediated, non-IgE-mediated, and mixed food allergic (FA) reactions [1]. In a broad term, FH also includes food pseudo-allergies (FPA), which are reactions that mimic FA, leading to the release of mediators, e.g., histamine but lack immunological mechanisms.

It should note that FA prevalence is between 5% and 10% throughout the developed world and has risen over recent decades [2]. Therefore, FA has become a significant public health burden. FAs are most common in children below three years of age but also occur in adults. Up to 8% of children in the United States are now believed to be affected by FA [3].

FAs comprise a spectrum of adverse immunological reactions to specific dietary antigens, usually proteins. IgE-mediated FA is now a well-described medical condition. There are robust clinical research programs and primary science data about this disorder’s immune and molecular mechanisms. In contrast, the current understanding of non-IgE-mediated and mixed allergies (e.g., eosinophilic esophagitis (EoE)) molecular mechanisms and immunopathology contains many gaps in knowledge. Moreover, FPA clinical symptoms are almost identical to FA, but the molecular mechanisms are different and poorly covered.

This review aims to provide a modern classification and identify the primary diseases of non-IgE-mediated/mixed FH reactions, delineate the distinctive molecular features, and discuss recent findings in the immunopathology of EoE that may become a basis to develop valid biomarkers and novel therapies for this disease.

## 2. Classification and a Brief Description of Food Hypersensitivity Types Based on Molecular Mechanisms

**Figure 1:**
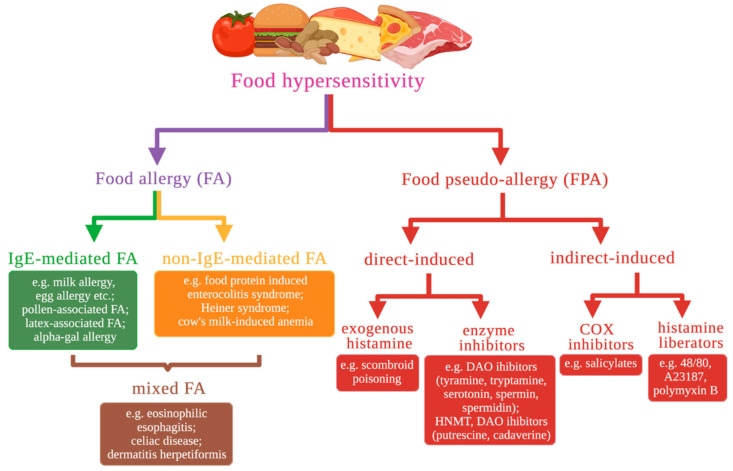
Classification of food hypersensitivity. Hypersensitivity reactions to food can be classified as food allergic (FA) or food pseudo-allergic (FPA) reactions. FA reactions are categorized further as IgE-mediated FA, non–IgE-mediated FA, or mixed FA (incl. eosinophilic esophagitis). FPA reactions are categorized further as direct-induced FPA or indirect-induced FPA. COX—cyclooxygenase, DAO—diamine oxidase, HNMT—histamine-N-methyltransferase.

FH is a broad term for an abnormal response related to food ingestion. Based on the pathophysiological mechanism of the reaction, food hypersensitivity can be divided into two broad categories [4]. The first category is immune-mediated reactions (i.e., FA). FA reactions are pathological immunologic responses to particular food antigens (called allergens) in a susceptible host. These reactions are reproducible each time the allergenic molecules (typically food protein antigens) are ingested. Based on the immunological mechanisms involved, FAs may be further classified into three types (Figure 1) [3].

IgE-mediated FA triggered when certain food allergen binds with allergen-specific antibodies that belong to the immunoglobulin E (IgE) class. It is the most common, best known, and well-characterized FA type. Typical IgE-mediated food allergic reactions occur immediately after allergen exposure, reproduce each time allergen is ingested, and are caused by food-specific IgEs, which can be detected using different approaches in order to diagnose FA and detect sensitization to specific allergen, i.e., persistence of allergen-specific IgEs [5]. This group includes milk allergy, egg allergy, pollen-associated FA, latex-associated FA, alpha-gal allergy, etc.

In sensitized individuals, allergen-specific IgE binds to FcƐRI with its Fc region. FcƐRI is the high-affinity IgE receptor. Mast cells, basophils, activated eosinophils, and some subtypes of antigen-presenting cells express FcƐRIα subunit on the surface of cellular membrane, allowing them to bind IgE molecules. To initiate downstream cellular signaling, multivalent allergen needs to bind with several IgEs-FcƐRIα complexes. Allergen initiates aggregation of IgEs-FcƐRIα recruits Lyn kinase, that phosphorylates FcεRIβ and γ subunits, allowing spleen tyrosine kinase (Syk) activation. Syk phosphorylates adaptor proteins, including linker for activation of T-cells (LAT). LAT recruits phospholipase PLCγ1, which produces second messengers: inositol 1,4,5-trisphosphate (IP3) and 2,3-diacylglycerol (DAG). IP3 activates exocytosis and degranulation of mast cells and basophils via increase of intracellular Ca^2+^ level. DAG activates protein kinase C, which phosphorylates myosin light chain to transport granules to cellular membrane. Mediators in mast cell and basophil granules (histamine, serotonin, serine proteases and others), when released, cause vasodilatation, smooth muscle constriction, increase vascular permeability, producing FA symptoms, e.g., urticaria, angioedema, diarrhea and vomiting, bronchospasm, hypotension [6].

In non-IgE FA, specific IgE to food antigens are not involved in an allergic reaction. Cellular mechanisms of the immune response and type II and III hypersensitivity are responsible for FA. Non-IgE FA includes various disorders such as food protein-induced enterocolitis syndrome (FPIES), Food Protein-Induced Allergic Proctocolitis (FPIAP), Heiner’s syndrome, cow’s milk-induced anemia, etc.

In mixed FA, both antigen-specific IgE and immune cells are involved in the reaction. Mixed FA has been increasing worldwide [7]. The most common mixed FAs are Eosinophilic Esophagitis (EoE) and Non-EoE eosinophilic gastrointestinal disorders (Non-EoE-EGID), which may occur as eosinophilic gastritis, colitis, or gastroenteritis.

The second category is FPA, which is similar to true allergies but differ from FA in that they are not a consequence of a dysregulation of the immune system. FPA occurs due to the properties of the food itself and the abnormal response of the host. The first may be due to components in food products that may be either exogenous or present naturally in food. Abnormal responses of the host include functional nontoxic and nonimmunologically mediated reactions. Natural and artificial organic compounds may cause adverse food reactions in sensitive people if consumed sufficiently; the degree of sensitivity varies between individuals.

FPA can be divided into two groups depending on the induction of histamine release. Direct-induced FPA is manifested by the action of exogenous histamine (from histamine-rich foods) [8] or enzyme inhibitors (tyramine, tryptamine, putrescine, etc.) that initiate the release of histamine from cells. Metabolism disorders are most commonly attributed to diamine oxidase (DAO) enzyme deficiency. Less common causes are histamine-N-methyltransferase (HNMT) and aldehyde oxidase (AOX1) deficiencies, which alter intracellular histamine breakdown.

Indirect-induced FPA is caused by foods containing histamine-releasing agents–COX inhibitors or histamine liberators. Salicylate–containing foods cause FPA reactions [9] based on the inhibition of cyclooxygenase–1 (COX1) by salicylates from natural food sources. COX1 inhibition results in reduced arachidonic acid use in the prostaglandin synthesis pathway. In intolerant individuals, this leads to activation of the leukotriene metabolism with increased formation of LTB4 and/or LTC4–E4. Typical symptoms of salicylate intolerance are respiratory complaints, including asthma and sinus inflammation with recurring nasal polyps, known as Samter’s Triad [10]. Sometimes, symptoms may include gastrointestinal complaints with meteorism, flatulence, diarrhea, and, rarely, colitis with strictures and ulcers [11]. Histamine liberators can induct release histamine from mast cells and eosinophils without binding to cell receptors. Compound 48/80, ionophore A23187 can cause significant histamine release. In each case, a release is triggered by an increase in levels of free cytosolic calcium [12]. However, the pathogenesis of the action of histamine liberators is even less clear.

## 3. Eosinophilic Esophagitis

Eosinophilic esophagitis (EoE) is a recently recognized allergic-mediated disease with eosinophil-predominant esophagus inflammation. Clinically, it is characterized by various symptoms related to esophageal dysfunction, including vomiting, regurgitation, feeding difficulties, heartburn, failure to thrive in infants, dysphagia, or food bolus impaction [13]. Symptoms are nonspecific and mimic those observed in gastroesophageal reflux disease (GERD).

Diagnosis is based on esophagogastroduodenoscopy (EGDS) and esophageal biopsy [14]. Macroscopically, EGDS can be normal or show signs of inflammation or fibrosis [15]. The disease leads to the development of patches with normal areas of mucosa mixed with inflamed ones [16]. Histopathological findings in biopsy include eosinophilia organization of eosinophils in microabscesses, lymphocytosis, dilation of intercellular spaces (DIS) in the esophageal epithelium (also termed spongiosis), epithelium basal zone hyperplasia, lamina propria papillae elongation, and fibrosis [17,18,19]. Diagnosis of EoE requires at least one esophageal biopsy with 15 or more eosinophils present in one high–power field (EOS/HPF) [14].

The disease occurs both in pediatric and adult populations and is especially common amongst males. Foods are the major antigenic trigger for EoE in children and adults. Milk, egg, wheat, and soy are the most common. The individuals affected by EoE have a high rate of atopic comorbidities (i.e., allergic rhinitis, asthma, IgE mediated FA, and/or eczema), with 28% to 86% of adults and 42% to 93% of children having another allergic disease [20]. In addition, high rates of IgE–mediated food allergy (15% to 43%) are present in the EoE–affected individuals, especially in children [21].

Despite the high rate of IgE-mediated atopic diseases, evidence suggests that IgE has no direct role in EoE pathogenesis. Immunoassays for quantification of food-specific IgE levels and skin prick test results do not have predictive value for identification of true EoE food triggers [22,23]. Moreover, oral allergen immunotherapy, which is used for allergen-specific desensitization in patients with IgE-mediated FA, can cause development of EoE in 2–5% patients with remission of IgE-mediated FA. Oral allergen immunotherapy aims to decrease levels of allergen-specific IgE and increase levels of IgG4, which binds to allergen epitopes thus blocking IgE binding and mast cell degranulation. Therefore, high food-specific IgG4 levels may have implications for EoE pathogenesis [24,25,26]. Children who outgrow IgE-mediated FA and reintroduce these foods in their diet can later develop EoE to the same food [27]. In animal models of experimental EoE, B cell–deficient mice still developed EoE without IgE [28]. Omalizumab, an anti–IgE monoclonal antibody used in IgE–mediated FA treatment, was ineffective in treating EoE [29,30].

Studying genetic variants in the EoE transcriptome provides a deep understanding of the molecular mechanisms of EoE.

### 3.1. The Role of the Eotaxin-3 and IL-13 in the Development of EoE

The *CCL26* gene has the largest fold change in mRNA expression level between EoE transcriptome and controls in many studies [31,32,33]. The *CCL26* codes chemokine Eotaxin-3, implicated in eosinophil trafficking to the esophagus in patients with EoE via chemokine receptor CCR3. Of the eotaxins, *CCL26*/Eotaxin-3 is the most upregulated in patients with EoE, and its expression correlates with eosinophil (and mast cell) levels within esophageal biopsy specimens, indicating a specific contribution in the disease. Only modest changes of other eotaxin family genes (*CCL11*/Eotaxin–1 and *CCL24*/Eotaxin-2) were observed in EoE patients. The mouse homolog of *CCL26* is a pseudogene [34], although CCR3–deficient mice were nearly wholly protected from the development of esophageal eosinophilia in the experimental EoE model [32]. Levels of *CCL26* transcript in a single biopsy specimen are susceptible in distinguishing EoE from control populations [35] and GERD patients [36], despite the histological “patchiness” of EoE across multiple biopsy specimens.

Studies have determined that TH2–derived interleukin IL–13 is one of the critical signaling molecules altering gene expression in EoE. It is well established that IL–13 is overproduced in EoE patients’ biopsy specimens. *IL–13* mRNA expression in active EoE by RT–PCR was 16–fold higher compared to healthy controls [33,35]. In contrast, the *IL–4* mRNA level was not significantly increased in EoE. Still, a statistically significant difference in IL–4 expression was observed between EoE patients with and without atopic comorbidities, with higher *IL–4* expression levels in atopic individuals [35].

The *CC10–rtTA–IL13*–transgenic mouse is a well–characterized model of asthma [37]. These mice contain transgenic construct that makes possible external regulation of *IL13* gene expression in lung tissue. *IL–13* overexpression with the *CC10–rtTA–IL13* transgenic system in response to exogenous doxycycline is sufficient to induce alteration resembling EoE, i.e., esophageal eosinophilia, tissue remodeling of the esophagus: increased esophageal circumference, increased epithelial cell proliferation primarily associated with the basal zone, collagen deposition, and increased angiogenesis in the lamina propria [38]. IL–13–induced changes in murine esophageal transcriptome significantly overlap with human EoE transcriptome data [38], including murine Eotaxin–1 and Eotaxin–2 esophageal production.

**Figure 2:**
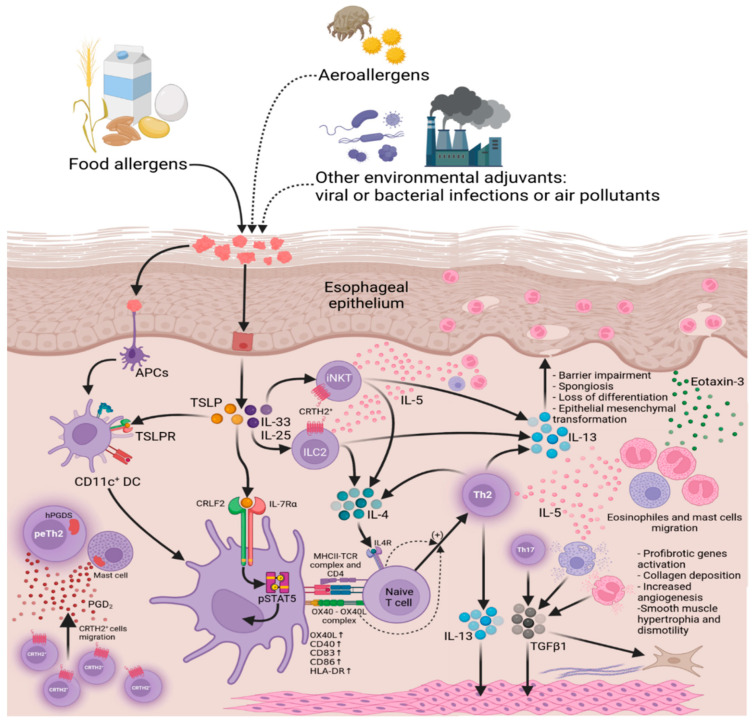
Cellular mechanisms of eosinophilic esophagitis pathogenesis. Allergens/adjuvants (incl. food allergens) stimulate the esophageal epithelium by inducing thymic stromal lymphopoietin (TSLP) and Interleukin (IL)–33, leading to stimulation of T helper 2 cells (Th2), natural killer cells (NK cells), mast cells, basophils, and type 2 innate lymphoid cells (ILC2). NK cells, mast cells, basophils, ILC2, and Th2 cells induce IL–4, which induces Th2 differentiation. IL-4 and IL-13 induced by Th2 cells provoke the release of Eotaxin–3, which stimulates eosinophils to secrete IL–5. IL–5 secreted by Th2 cells and mast cells also stimulate eosinophils. Mast cells, eosinophils, Th2 cells induce transforming growth factor beta 1 (TGF*β*1), stimulating eosinophils and fibroblasts. Th2 cells also induce IL–13, which causes impaired barrier function and tissue alteration. APCs—antigen-presenting cells, TSLPR—thymic stromal lymphopoietin receptor, DC—dendritic cells, peTh2—pathogenic effector Th2 cells, CRTH2—prostaglandin D2 receptor 2, hPGDS—human prostaglandin D synthase, CRLF2—cytokine receptor-like factor 2, pSTAT5—phosphorylated signal transducer and activator of transcription 5, MHCII—major histocompatibility complex class II, TCR—T-cell receptor, OX40L—ligand for OX40, iNKT—invariant natural killer T-cells.

Esophageal epithelial cells express all components of the IL–13 receptor, including IL–4Rα, IL–13Rα1, and IL–13Rα2 [39]. IL–13 is produced by Th2 cells [40], activated eosinophils [41], ILC2, and iNKT cells (Figure 2).

In situ hybridization on esophageal biopsy specimens identified the esophageal epithelium as the main source of Eotaxin–3 production [32]. Primary esophageal epithelial cells stimulated with IL–13 produced transcriptional changes largely overlapped with the EoE transcriptome (22% of IL–13–induced genes were present in the EoE transcriptome) [33]. *CCL26* was the most upregulated gene in the IL–13–stimulated esophageal cells [33]. IL–13 and IL–4 activate signal transducer and activator of transcription 6 (*STAT6*) [39]. The STAT6 binding site (–55 to –64) is located upstream of the *CCL26* transcription initiation site and is required for IL–13–induced *CCL26* promoter activity in esophageal epithelial cells [33,42]. In addition, the cAMP-response element (CRE) site in the *CCL26* promoter (–230 to –237) acts in concert with the STAT6 site [42] and functions as a transcriptional coactivator for STAT6 [43]. ChIP analysis has shown that STAT6 binds to the CCL26 promoter and recruits CRE-binding protein (CREB) binding protein (CBP) following stimulation with IL–13 [42]. CBP activates basal and IL–13–induced *CCL26* promoter activity. CBP acetylates histone protein H3 at the transcription start site (TSS) and promotes an open chromatin structure facilitating *CCL26* transcription [42]. Moreover, higher levels of H3 acetylation were observed in the esophageal tissue in EoE compared to the control, which may be partly attributed to the IL–13–dependent activation of CBP intrinsic histone acetyltransferase activity [42].

### 3.2. Impairment of Esophageal Epithelium Barrier Function

The prominent pathophysiological feature of EoE is impairment of esophageal epithelium barrier function (BF). The healthy esophageal epithelium protects against adverse environmental factors, including food antigens and gastric acid refluxate, that are able to penetrate into esophageal tissue causing structural damage or inflammatory responses. The vast body of evidence suggests that barrier dysfunction and allergic inflammation-related changes in the esophageal epithelium are essential processes in EoE pathology.

The morphology of the inflamed esophageal epithelium in EoE has several features, including basal zone hyperplasia in the esophageal epithelium, which replaces much of the more differentiated upper layer of epithelial cells, and the emergence of DIS in the suprabasal layers, which is believed to be associated with an increase in permeability of esophageal epithelium to food allergens, refluxed acid, microbes, and other alternating factors in EoE.

Impaired BF of the esophageal epithelium in EoE patients has multiple acknowledgments, including ex vivo assays, inactive EoE biopsies, and functional tests in patients. There are some methods to test epithelial BF: a measurement of transepithelial electrical resistance (TER) in biopsy specimens or air-liquid interface (ALI) cell cultures, mounted in Ussing chambers, and electrical tissue impedance spectroscopy (ETIS).

In EoE patients, TER and mucosal impedance are significantly lower and mucosal permeability is higher than in healthy control groups. Additionally, endoscopic mucosal ETIS can predict EoE activity.

Fluorescein isothiocyanate-labeled dextran (FITC–dextran) is a common marker for epithelial permeability assays. In this assay, FITC–dextran flux through the sample is measured by its fluorescent signal enabling evaluation of epithelial BF and permeability.

Importantly, these morphological and functional changes are reproduced in the ALI cultures of esophageal epithelium differentiated in the presence of IL–13. IL–13 leads to disruption of esophageal epithelium cell architecture and impairs its BF [44].

On the molecular level, epithelial BF depends on the proper expression of structural genes coding proteins that comprise multiprotein complexes called cell junctions. Cell junctions provide adhesion between two cells (e.g., between neighboring epitheliocytes in esophageal epithelium) or cell and extracellular matrix proteins. Cell junctions are involved in intracellular signaling. Cell junction proteins’ dysregulation leads to loss of adhesion between cells impairing epithelial BF and increasing paracellular permeability, and at the same time dysregulates signal transduction in the cells. Proteins of tight junctions (claudins 1 and 7 and occludin) [45,46,47], adherens junctions (E–cadherin), and desmosomes (*DSG1*) [44,45] are shown to be downregulated in EoE.

### 3.3. The Role of the Cadherin 26 in the Development of EoE

However, the recently characterized cadherin 26 (*CDH26*) is highly upregulated in both active EoE [33] and eosinophilic gastritis (EG) [48,49] patients. It was the only intersecting upregulated gene in these data. *CDH26* gene expression is upregulated in epithelial cells by Th2 cytokines [49]. Immunohistochemical staining of control group biopsies with CDH26-specific antibodies revealed that *CDH26* expressed in superficial layers of esophageal epithelium. Staining in active EoE specimens covered both epithelial cells of superficial layers and basal zone cells. Intensity of CDH26 staining in active EoE biopsies surpassed control levels of *CDH26* [49]. By Western blot analysis, CDH26 had a 4.9–fold increase in EG and 3.4–fold increase in EoE compared to the control, so CDH26 is highly upregulated in esophageal and gastric tissues under allergic inflammation [49]. CDH26 exhibits sequence homology to the cadherin family of proteins, with five extracellular cadherin repeats [50]. *CDH26* has been shown to localize on the cell surface membrane of esophageal epithelial cells and be modified by N–linked glycosylation of asparagine residues [49]. Co–immunoprecipitation shows that *CDH26* is a functional cadherin that interacts in a homotypic manner with other *CDH26* molecules, mediates calcium–dependent cell adhesion, dimerizes or multimerizes, and interacts with *α*–, *β–*, and p120–catenins [49,51]. CDH26 also binds *α*4 and *α*E integrins that are co-immunoprecipitated with CDH26 [49]. The recombinant CDH26–hIgG1–Fc antibody binds *α*4*β*7 integrin, CDH26–expressing cells adhere to integrin *α*4*β*7–coated surface, and Jurkat cells that express integrin *α*4*β*1 [52] adhere to recombinant CDH26–hIgG1–Fc in an integrin *α*4–dependent manner, so, CDH26 is proposed to have the ability to impact diverse *α*4+ and/or αE+ cells (e.g., CD4+ T cells, eosinophils, and mast cells) migration and adhesion [49]. Altered intraepithelial localization of several subsets of cells in EoE correlates with this fact.

However, *CDH26* modulatory action on leukocyte activation is less clear. It was expected that *CDH26* would be a CD4+ T–cell co-stimulatory molecule, similar to other *α*4*β*1 ligands including fibronectin [53] and VCAM-1 [54,55]. However, peripheral blood CD4+ T cells in TCR suboptimal stimulation conditions showed attenuation of *CD25* expression (a marker of activated T–cells, IL–2R*α*) when co-incubated with *CDH26*–hIgG1–Fc (but not with normal IgG) [49]. *CDH1*–hIgG1–Fc also has been shown to attenuate CD25 expression in these experimental conditions [49]. Cadherin–Fc constructs also inhibited the secretion of IL–2 in response to TCR stimulation in CD4+ T–cells [49]. So, *CDH26* and *CDH1* may have co-inhibitory action on CD4+ T-cells. Although CDH1 (also known as epithelial (E)–cadherin) has previously been shown to co-stimulate CD4+ T cell activation [56], in some conditions, it can inhibit activation of some T– and other immune cell subsets, i.e., ligation of CDH1 expressed by murine epidermal *γδ* T cells, called dendritic epidermal T cells (DETC), with CDH1 expressed by epidermal keratinocytes inhibits TCR-dependent DETC activation, cytokines production (IFN–*γ*, TNF–*α*), and degranulation [57]. Moreover, CDH1 is known to be a counterreceptor for killer cell lectin–like receptor G1 (*KLRG1*) of NK cells, memory T cells, and type 2 innate lymphoid cells (ILC2) [58,59,60]. It can be hypothesized that the CDH26 increase in active EoE and EG is involved in both promoting diseases, i.e., by increasing migration of some cell types that occur in inflamed mucosa and epithelium in EoE or by some other ways, and resolution of inflammation, inhibiting and dampening Th2–mediated activation to promote the return of the tissue to homeostasis (similar to CAPN14 mode of action in epithelial cells, see further). Alternatively, it is possible that CDH26 inhibits only particular subsets of CD4+ T cells, and depending on the inhibited cell subset, CDH26 can either facilitate or dampen Th2–mediated allergic inflammation [49]. Another interesting finding in this research is that CDH26-hIgG1–Fc (as well as CDH1–hIgG1–Fc) has immunosuppressive potential in CD4+ T cells and can be used as a novel treatment strategy in EoE, EG, or some other diseases [49]. Additional research is needed to explain CDH26 function in EoE and EG pathogenesis.

### 3.4. The Role of the Desmosomal Cadherin Desmoglein-1 in the Development of EoE

The desmosomal cadherin desmoglein–1 (*DSG1*) is one of cell adhesion molecules, glycoprotein assigned to the cadherin superfamily. *DSG1* is an essential component of desmosomes, and forms cell-to-cell junctions in epithelia, e.g., in epidermis. *DSG1* is considered to be involved in pathogenesis of atopic diseases, e.g., homozygous mutations in *DSG1* causes severe dermatitis, multiple allergies, and metabolic wasting (SAM syndrome); autoimmunization against *DSG1* causes pemphigus foliaceus, a skin blistering disease, which manifests as severe loss of epithelial integrity, compromised BF and skin lessions [44]. There is a substantial decrease (12.7–fold reduction in RNA–seq and 22.1–fold reduction in RT–PCR) in the expression of *DSG1* in the esophageal biopsies of patients with active EoE [44]. The downregulation of *DSG1* was specific among other *DSG* family members, including *DSG3*, the most abundant in esophageal mucosa DSG. Immunofluorescent and immunohistochemical staining for *DSG1* revealed that expression of this protein is mainly localised to suprabasal layers of esophageal epithelium in control biopsy samples. In active EoE, pronounced loss of *DSG1* expression was observed [44]. *DSG3* and E–cadherin were unchanged between the control and active EoE in this study [44]. The reduction in *DSG1* levels is consistent with a significant decrease in the number of desmosomes per cell, which is a distinctive ultrastructural feature of active EoE compared with inactive EoE, GERD, and normal epithelia [61].

*DSG1* gene silencing was performed on EPC2 ALI culture. Cells were transduced with small hairpin RNA (shRNA) targeting *DSG1* using a lentiviral vector in order to directly examine the impact of *DSG1* downregulation on esophageal epithelial cell adhesion. Prominent spongiosis was observed in the suprabasal layers of cells transduced with shRNA to *DSG1* [44]. Electron microscopy showed prominent alterations in the epithelium ultrastructure in active EoE biopsies compared to control. Dilated intercellular spaces were observed in EoE patients’ epithelium instead of the cohesive intact epithelia in controls [44,61]. The dispase adhesion assay has shown significantly greater cell dissociation in DSG1–deficient cells compared to controls [44], suggesting a decrease in esophageal epithelial cell adhesion and highlighting the essential role of *DSG1* for esophageal epithelium integrity. These facts suggest that impairment of BF in EoE may be caused by other alterations in cellular contacts, e.g., observed in the EoE loss of *DSG1*. Experiments with *DSG1* gene shRNA silencing in ALI–differentiated EPC2 cells reproduced this impairment in BF. shRNA–transduced cultures demonstrated impaired TER (42% decrease) and increased FITC–dextran paracellular flux (33%) [44]. So, *DSG1* loss is sufficient to impair esophageal epithelium BF in vitro, and *DSG1* mRNA and protein decrease in patient biopsies has a significant contribution to BF impairment in EoE [44].

The effects of IL–13 on the integrity and barrier formation of ALI–differentiated esophageal epithelial cells were examined. IL–13 significantly downregulated *DSG1* induction in ALI–differentiated cells at a concentration of 100 ng/mL, whereas induction of *KRT10* and *DSG3* during ALI differentiation was not affected. IL–13 promotes the impairment of esophageal epithelium BF. At both analyzed concentrations of IL–13 (10 or 100 ng/mL), the IL–13–induced phenotype of ALI–differentiated EPC2 cells was represented by separation of suprabasal layer cells (spongiosis), which reflects the phenotype of DSG1–deficient cells and indicates the impairment of BF. IL–13 promoted the impairment of esophageal epithelium BF and was also documented by a significant reduction in TER at both the third and fifth day after treatment with IL–13 (100 ng/mL).

IL–13–dependent *DSG1* downregulation was shown in vivo using the CC10–rtTA-IL13 murine model. Overexpression of IL–13 in doxycycline-treated mice reduced *DSG1* mRNA and protein levels in the esophageal mucosa as compared with untreated mice.

### 3.5. Loss of Esophageal Epithelium Differentiation

IL–13 contribution to EoE pathogenesis beyond Eotaxin–3 overproduction includes profound dysregulation of the epidermal differentiation complex (*EDC*) gene expression [62]. The epidermal differentiation complex (*EDC*) is a gene complex on the human chromosome 1 in a locus *1q21*. Genes residing in *EDC* have similar, closely related functions and are essential for epithelial barrier formation and expressed during maturation of epithelial cells terminal differentiation [63]. Across the human genome, the highest density of genes, expression of which is dysregulated in active EoE, is observed to occur in *EDC* locus [62]. Expression patterns in esophageal biopsy specimens of EoE patients show significant decreased expression or trends toward the decreased expression of most genes in the EDC locus [62]. Ex vivo response to IL–13 presents a similar downregulation of EDC genes, including filaggrin (*FLG*), involucrin (*IVL*), and several small proline–rich repeat (*SPRR*) family members (1A, 2D, 3, and 4) [62]. *FLG* is expressed in the skin epidermis and epithelium of esophageal, nasal and oral mucous membranes. *FLG* encodes progenitor protein Profilaggrin. During epithelial cell differentiation, Profilaggrin undergoes processing, and after proteolytic cleavage Filaggrin monomers are formed. Filaggrin is one of the essential structural proteins for stratified epithelial BFs. Filaggrin function is aggregation of keratin intermediate filaments during transformation of granular cells into flattened squamous cells, that compose the superficial layer of esophageal epithelium and essential for its BF, despite the fact that actual keratinization normally does not occur in esophagus, and human esophageal epithelium is stratified, squamous and nonkeratinized [64]. In the case of *FLG* downregulation, epithelial BF decreases, and exogenous allergens become able to penetrate epithelial barriers and activate immune responses. Loss of *FLG* and other *EDC* gene expression leads to defects in epidermal BF. *FLG* and *IVL* expression in EoE biopsies is decreased on gene and protein level [65]. IL-13 also decreases levels of *FLG* and *IVL* mRNAs and proteins in ALI-cultured primary human esophageal epithelial cells (HEEC) [65]. Furthermore, *FLG* silencing with siRNA in ALI HEEC causes BF impairment; TEER and thickness of the cell layer was decreased in siRNA-transfected cultures, indicating alterations in cell proliferation and differentiation [65]. It is remarkable that tight junction proteins (*CLDN1* and *CLDN4*) have altered patterns of expression in *FLG*-deficient cells, although the levels of the proteins are unchanged [65].

There is a known association between *FLG* loss–of–function (LOF) mutations and predisposition to atopic dermatitis (AD) [65] and enhanced percutaneous sensitization in IgE–mediated allergies [66]. The EoE population has high rates of atopic diseases, and *FLG* LOF mutations are common in EoE patients [62].

Molecular mechanisms implicated in the loss of esophageal epithelium differentiation in EoE were further investigated in tissue–specific esophageal genes expression data from RNA–seq [67]. *CAPN14* and *SERPINB13* were two tissue–specific esophageal genes significantly upregulated in EoE patients [67]. Functional enrichment gene ontology identified endopeptidase inhibitor activity and keratinization as the most profoundly dysregulated molecular functions and biological processes. The number of *SERPIN*–related genes was upregulated (*SERPINB2*, *SERPINB3*, *SERPINB4*, and *SERPINB13*), whereas genes from the serine protease inhibitor Kazal–type (*SPINK*) family were downregulated (*SPINK5*, *SPINK7*, and *SPINK8*) [67]. Increased expression of SERPINE1 (*plasminogen activator inhibitor type I, PAI–1*) has been previously reported in patients with EoE [68]. The downregulated genes included epithelial differentiation markers KRT6B, IVL, and SPRR proteins [67]. Transglutaminases crosslink cornified envelope (CE) precursors (i.e., loricrin, involucrin, envoplakin, and periplakin [69]) in keratinocytes during their differentiation in order to form highly insoluble CE [70]. *TGM1* and *TGM3* were also downregulated in EoE [67]. These findings revealed a profound loss of esophageal tissue differentiation in patients with EoE. Expression patterns of tissue–specific esophageal genes from biopsy specimens of patients with active EoE and in ALI cell cultures differentiated in the presence of IL–13 were remarkably similar in this study [67].

### 3.6. The Role of the CAPN14 in the Development of EoE

CAPN14 is a cytosolic calcium–activated cysteine protease that was identified as an associated locus 2p23 in EoE genome-wide association studies (GWAS) [71,72]. In comparison with other members of the *calpain* family, *CAPN14* possesses a unique feature of its tissue-specific expression pattern. *CAPN14* is almost specifically expressed in the esophageal epithelium [72]. Stimulation of EPC2 esophageal epithelial cells with IL-13 significantly upregulates *CAPN14* expression. IL-13 impact on calpain family gene expression is confined to *CAPN14* upregulation; other calpains except *CAPN14* are not induced by IL-13 in primary esophageal epithelial cell culture and in EPC2 ALI cultures [72,73]. However, in specimens of esophageal biopsies, in addition to *CAPN14* upregulation, *CAPN3* level turned out to be significantly elevated. Conversely, expression of *CAPN7*, *CAPN5*, *CAPNS2* and *CAST* (*calpastatin, endogenous calpain inhibitor*) genes were downregulated [72]. Throughout the calpain family and related genes, *CAPN14* reveals highest fold change.

The kinetics of IL-13-induced *CAPN14* expression are parallel to the induction of *CCL26* in EPC2 cells [74]. Pronounced changes in an epigenetic signature are observed in the promoter region of *CAPN14* in response to IL–13 stimulation. The ChIP–seq detected a marked increase in H3 acetylation at the 27th lysine residue (*H3K27Ac*) [72,75] and H3 trimethylation at the 4th lysine residue (*H3K4me3*) [75] in the *CAPN14* promoter region near the TSS in IL–13–treated cells. *H3K27Ac* and *H3K4me3* are highly enriched at active promoters near TSS and positively correlated to gene transcription, which is consistent with an increase in *CAPN14* transcriptional activity by RNA–seq [72].

Similar to the STAT6-dependent *CCL26* gene, IL–13 or IL–4 exposure is sufficient to upregulate *CAPN14* expression. Esophageal epithelial cells transfected with nanoluciferase-expressing reporter constructs with either *CCL26* or *CAPN14* promoters exhibited a similar upregulation of the nanoluciferase signal in IL–13 or IL-4 stimulation [75]. These data lead to the assumption that *CAPN14* expression is *STAT6*-dependent. Based upon the *STAT6* canonical core motif, two putative *STAT6*-binding sequences were identified within 90 base pairs of the TSS in the *CAPN14* gene promoter [75]. The third *STAT6*-binding site was located at the first *CAPN14* intron [75]. Site-directed mutagenesis was performed in the reporter construct with *CAPN14* promoter in order to evaluate the contribution of each putative *STAT6* site to promoter activity. Mutation of the distal *STAT6* site attenuated IL–13–mediated induction of *CAPN14* promoter activity, while mutation of the second STAT6 site completely blocked *CAPN14* promoter induction [75]. The third putative STAT6 site did not affect the IL–13–induced activity of the *CAPN14* promoter [75]. Chromatin was extracted from esophageal epithelial cells stimulated with IL–13, and ChIP–seq with anti–STAT6 antibodies was performed. Peaks spanning two *STAT6* sites in the *CAPN14* promoter were identified [75]. The intronic putative site produced no read peaks. Hence, it lacked *STAT6* binding [75]. Moreover, the ChIP–seq data analysis showed that STAT6 peaks overlapped peaks from the active histone marks *H3K4me3* and *H3K27Ac* appearing after IL–13 treatment [75]. This evidence suggests that IL–13 or IL–4 upregulates *CAPN14* expression through *STAT6* activation and its binding to identified *STAT6* sites in the *CAPN14* promoter region. Each of two *STAT6*–binding sites is essential for full IL–13–induced *CAPN14* upregulation.

In GAWS, the variants most highly associated with increased risk of EoE were found at *2p23* spanning the *CAPN14* gene. Primarily, the single nucleotide polymorphism (SNP) *rs77569859* was identified as most highly associated with EoE. The EoE–risk haplotype at *2p23* includes 12 variants in linkage disequilibrium (r2 > 0.8). Six SNPs most highly associated with EoE were identified at the *CAPN14* locus after imputation with a composite reference panel of integrated haplotypes from the 1000 Genomes Project. Two SNPs (*rs76562819* and *rs75960361*) were located in putative regulatory regions and overlapped with *IL–13*–induced *H3K27Ac* peaks on ChIP–seq. *rs76562819* is located proximal to the 5′ of the *CAPN14* TSS, within 45 bp from the distal *STAT6* binding site, intersects a *H3K4me1*–enriched region, and lies at the open chromatin region, which were identified in publically available ENCODE functional genomics data from ChIP–seq and DNaseI hypersensitivity site mapping assays. An electrophoretic mobility shift assay (EMSA) with oligonucleotide probes containing the risk (G) or non-risk (A) allele of *rs7656219* revealed that the promoter region around identified SNP binds nuclear factors in a genotype–dependent manner, with the risk allele preferentially binding to a nuclear protein complex that is present in IL–13–stimulated esophageal epithelial cells [72]. Surprisingly, *CAPN14* realtime PCR expression in EoE patients’ biopsies with risk haplotype at *2p23* (i.e., having at least one of the EoE risk alleles at each of the six most highly associated variant locations) was 30% lower than in EoE patients with the non-risk haplotype (without EoE risk alleles). Among variants of the EoE risk haplotype *rs7656219* has genomic evidence supporting its specific role in the promoter activity of *CAPN14*. To examine whether *rs76562819* is sufficient to result in genotype–dependent promoter activity of *CAPN14*, two luciferase reporter constructs with the *CAPN14* promoter and first intron fragment were used. The construct containing non–risk allele of *rs76562819* was obtained by site–directed mutagenesis in the risk allele-containing construct. The EoE risk allele at *rs76562819* resulted in a 40% reduction in IL–13 and IL–4–induced *CAPN14* promoter activity compared to the EoE non–risk allele, which is consistent with *2p23* haplotype–dependent expression in EoE biopsies. The reporters did not demonstrate a statistically significant difference in genotype–dependent promoter activity without IL–13 stimulation. The reporter constructs assay revealed that the *rs76562819* genetic variant was sufficient to produce genotype-dependent promoter activity highly similar to what is observed between risk and non–risk haplotypes in EoE patients, so it can be concluded that the *rs76562819* EoE risk allele results in the decrease in IL13–induced *CAPN14* expression in EoE patients.

Two major isoforms of *CAPN14* mRNA are identified. Differential isoform usage analysis revealed no difference in proportion of *CAPN14* isoforms between EoE and control, regardless of sex [75]. Of the two common *CAPN14* isoforms, expressed in humans, *ENST00000403897* (*ENST00000444918*) is more prevalent and includes *exon 7*. Isoform *ENST00000398824* has lower expression level, lacks the *exon 7* of *CAPN14* gene, and is predicted to undergo nonsense-mediated decay (NMD), which can explain lower prevalence of second isoform [75].

*CAPN14* overexpression assays suggest that an elevated level of *CAPN14*, similar to IL–13–induced expression level, is sufficient to cause disruption of the epithelial cell architecture, including separation of neighboring cells (acantholysis), separation of the epithelium from its underlying substrate (epidermolysis), and intraepidermal clefting (spongiosis) [74]. *CAPN14* overexpression significantly impairs epithelial BF. ALI cultures overexpressing *CAPN14* had 2.1–fold decreased TER and 2.6–fold increased FITC–dextran flux through the epithelial layer [74].

As previously mentioned, IL–13 induces *CAPN14* expression and downregulates *DSG1* on mRNA and protein levels. It has been shown that *CAPN14* overexpression in EPC2 ALI cell culture disrupts DSG1 protein integrity [74]. A specific decrease in *DSG1* expression was found on Western blot with anti–*DSG1* antibody. Moreover, a band with a lower molecular weight (50 kDa) appeared in *CAPN14* overexpressing samples but not in the empty vector controls [74]. The observed effects were specific to *CAPN14* activity; *CAPN1* overexpression did not produce an effect on the DSG1 level. The 50–kDa DSG1 band seen with *CAPN14* overexpression could also be induced with IL-13 stimulation [74]. Importantly, *CAPN14* gene silencing with shRNA targeting the 3′ UTR of *CAPN14* blocks the IL–13–mediated appearance of the 50–kDa DSG1 band [74]. DSG1 immunofluorescence staining was decreased by IL–13 stimulation, but this effect was partially rescued by *CAPN14* gene silencing. Remarkably, immunofluorescent signals of *CAPN14* and *DSG1* showed an inverse localization relationship in EPC2 ALI cell cultures [74]. This evidence suggests that *CAPN14* has a specific role in downregulating *DSG1* expression. Western blot results allow speculation that *DSG1* may be cleaved by *CAPN14* proteolytic activity. *DSG1* is not the only epithelial differentiation gene product found to be affected by *CAPN14* expression. Western blot analysis in *CAPN14*–overexpressing cells showed a decrease in filaggrin and profilaggrin, other proteins essential for epithelial BF [74]. All these findings indicate that IL–13–induced *CAPN14* activity impairs the BF of esophageal epithelium, but also increase in *CAPN14* activity affects a differentiation program of epithelial cells.

At the same time, however, *CAPN14* gene silencing dysregulates the IL–13–induced epithelial changes in ALI cultures. *CAPN14* gene silencing showed sustained BF and architectural changes after IL–13 stimulation compared with the nonsilencing control. Still, gene silencing increased the disorder of IL–13–mediated epithelial changes, including dilated intercellular spaces (5.5–fold area increase) and disruption of basal cell organization (1.5–fold decrease number of nuclei lining the basolateral edge) [74]. So, *CAPN14* involvement in IL–13–induced responses is not simply linear. *CAPN14* may be involved in either IL–13–induced epithelial barrier disruption or restoration of epithelial architecture, disordered by IL–13 [73]. Upregulation of *CAPN14* is linked to impairment of the epithelial BF, whereas its downregulation leads to failure in the repair of IL–13–induced epithelial changes [73].

*CAPN14* contribution to the restoration of IL–13–induced alterations of epithelial architecture may mechanistically explain why *rs76562819* SNP, associated with a remarkable decrease in *CAPN14*, simultaneously increases the risk of EoE.

### 3.7. The Role of the POSTN in the Development of EoE

**Figure 3:**
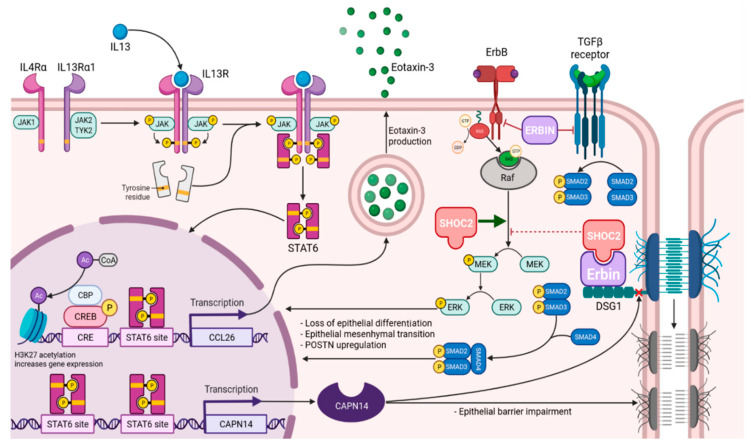
The signaling pathways of the interleukin–13 receptor (IL13R), transforming growth factor beta (TGF*β*) receptor, and epidermal growth factor receptor (ErbB) in esophagus keratinocyte and their alterations in eosinophilic esophagitis. The IL–13R receptor binds to its corresponding ligand, and heterodimerization occurs, enhancing Janus kinase (JAK) activity. Signaling molecules such as signal transducer and transcriptional activator (STAT) 6 and STAT3 can initiate transcription of target genes, including eotaxin–3. The effects of IL–13 are mediated by ErbB. ERBB2–Erbb2 interacting protein (ERBIN) negatively regulates TGF*β* signaling. TGF*β* mediates fibrosis by inducing fibrogenic target genes. Active TGF*β* binds to its receptor to initiate SMAD–dependent and independent signaling. SMAD–dependent signaling regulates fibrogenic target genes such as *α*–smooth muscle actin, collagen, connective tissue growth factor, tissue metalloprotease inhibitor, and periostin. TYK2—non-receptor tyrosine-protein kinase, CRE—cAMP response element, CREB—cAMP response element-binding protein, CBP—CREB-binding protein, CoA—Coenzyme A, Ac—acyl group, H3K27ac—lysine acetylation at N-terminal position 27 of histone H3, CCL26—chemokine (C-C motif) ligand 26, CAPN14—calpain-14, POSTN—periostin, DSG1—desmoglein 1, SHOC2—Leucine-rich repeat protein SHOC-2, ErbB—receptor tyrosine-protein kinase ErbB, TGF*β*—transforming growth factor *β*, MEK—mitogen-activated protein kinase kinase, ERK—extracellular signal-regulated kinase.

Moreover, DSG1 deficiency increases gene expression of the proinflammatory extracellular matrix molecule periostin (*POSTN*) [76]. *POSTN* is one of the markedly upregulated genes in EoE transcriptome (35-fold change), that encodes periostin—protein of extracellular matrix (ECM), that facilitates epithelial-mesenchymal transition (EMT), fibrotic remodeling and migration of certain cells to inflamed tissues. Periostin can directly enhance activated eosinophil adhesion via integrin αMβ2 [77], as well as increase keratinocyte production of thymic stromal lymphopoietin (*TSLP*), a potent Th2–skewing cytokine [78] that has been associated with EoE [79,80]. It has been shown that treatment of esophageal epithelial cells with IL–13 induces *POSTN* expression in this cell type [81], as well as in bronchial epithelial cells [82]. *POSTN* can induce epithelial–mesenchymal transition by increasing signaling through integrin *α*V*β*5 and epidermal growth factor receptor (*EGFR*) [83]. It is possible because epitheliocytes adopt a fibroblast-like phenotype due to induced loss of epithelial cell markers [84]. Moreover, it has been demonstrated that *DSG1*–dependent *EGFR* signaling suppression promotes epithelial differentiation and reduces proliferative capacity [85]. The epithelial–mesenchymal transition has been proposed to occur in EoE (Figure 3) [86,87].

### 3.8. EoE-Associated Risk Genes

Several different studies, including candidate-gene identification and genome–wide association studies (GWAS), have identified multiple genetic risk loci that are likely contributing to the development of EoE.

The first reported genome–wide significant EoE risk locus was the 5q22.1 containing *TSLP* gene. This locus was initially identified in 2010 by Rothenberg et al. [79], and two follow–up GWAS in 2014 confirmed 5q22.1 as an EoE risk locus [71,72].

In EoE, when allergens get to the esophagus and esophageal epithelium and allergic inflammation occurs, esophageal epithelium and other cells types secret various cytokines. *TSLP* is one of cytokines, produced by esophageal epithelium cells [88]. The main role of *TSLP* is regulation of Th cell phenotypes and promotion of their differentiation to Th2 cells by dendritic cells (DCs) and other APCs [78]. Known SNP in the *TSLP* gene augment the Th2 response, and TSLP levels are significantly higher in patients with atopic diseases and EoE [89].

Another genome–wide significant locus was reported in 2014. Two GWAS performed by Kottyan et al. and Sleiman et al. revealed a significant signal at the 2p23.1 locus (*CAPN14*) [71,72]. Further studies discovered prominent upregulation of *CAPN14* in EoE and tissue–specific expression of this gene in esophageal epithelium cells. Genetic and epigenetic mechanisms of *CAPN14* level regulation have been clarified, and the contribution of this risk gene to the molecular pathology of EoE was suggested (see Section 3.6 above).

In addition, the *11q13.5* locus passed the threshold of genome–wide significance in the GWAS by Sleiman et al. [71]. In the Kottyan et al. GWAS report, this locus had not reached genome–wide significance but was close to the threshold level. There are two relevant genes mapped to this locus: *LRRC32* and *C11orf30*, also known as *EMSY*.

*LRRC32* encodes a TGF–*β* binding protein, and *C11orf30* encodes a protein named EMSY involved in transcriptional regulation. *EMSY* and *LRRC32* are both expressed in esophageal epithelial cells. However, the exact mechanism of dysregulation in cellular signaling involving these proteins and the contribution of variations in these genes in EoE emergence and progression have not been reported yet [90].

For the recent GWAS reported in 2019 by Kottyan et al., the Immunochip platform was used. A previously not reported among EoE–associated locus was discovered at 16p13.13 with risk gene *CLEC16A*. However, *CLEC16A* has been associated with asthma, type 1 diabetes mellitus, and some other autoimmune diseases in the previously reported GWAS [91,92]. In the most recent GWAS of EoE risk genes reported in 2021 by Chang et al. [93], the 16p13.13 locus (*CLEC16A*) was replicated using the universal Illumina SNParrays.

The newly detected loci in this GWAS were at *5q31.1* (*RAD50*), *15q22.2* (*RORA*), and *15q23* (*SMAD3*). These genes have been associated with allergic diseases in previous GWAS [94], but the odds ratio (OR) for EoE is much higher for genes from this group, as compared with other allergic diseases. The OR difference suggests that there is a specific role of these new loci in EoE pathogenesis rather than in other allergic disorders.

**Figure 4:**
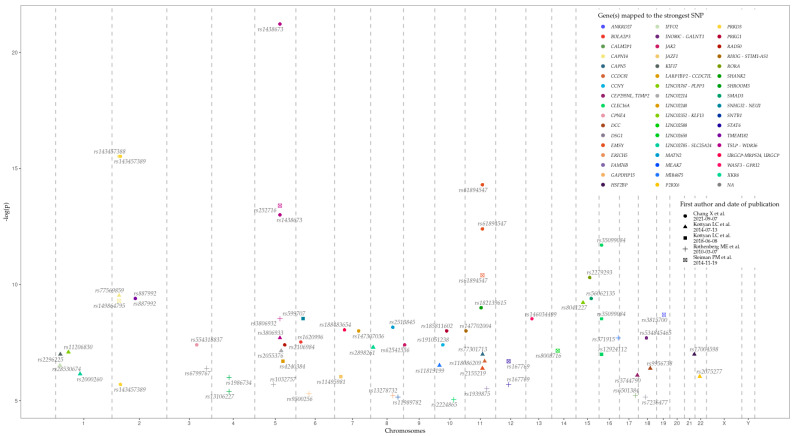
The position of the single nucleotide polymorphisms (SNP) with the most significant association with EoE from the genome-wide association studies (GWAS) data on the human genome.

Loci, genes, and SNPs that have the most significant association with EoE on the basis of GWAS data are represented in Table 1 and Figure 4. Data were retrieved from the GWAS Catalog (EFO ID: EFO_0004232). The biological meaning and role in EoE predisposition and development of many genes, represented in the table, remain obscure. Further studies are needed to identify the influence of associated variations, genes, and their products, on EoE pathogenesis.

## 4. Conclusions

EoE pathogenesis is a complicated network of interactions and signaling between epithelial, mesenchymal, and immune cells on molecular and intercellular levels. Alterations produced by overactivation of some cytokine signaling pathways, e.g., IL–13 or TSLP, were evolved and observed in this review from the viewpoints of molecular, genetic, epigenetic, and transcriptomic changes. Despite the substantial amount of experimental data, the reliable and representative mechanism of EoE pathogenesis has yet to show itself, and so the place of EoE between mixed and non-IgE-mediated allergic disorders, between eosinophilic gastrointestinal disorders currently seems vague and unclear.

## Figures and Tables

**Table 1:** Genome–wide significant loci reported in GWAS.

EoE Risk Locus	Mapped Gene	Tag SNP	The Strongest SNP Risk Allele	*p*-Value	OR	Reference
*1p13.3*	*LINC02785* *SLC25A24*	*rs2000260*	A	7 × 10^−7^	1.32	[72]
*1p32.2*	*LINC01767* *PLPP3*	*rs11206830*	?	8 × 10^−8^	2.162	[72]
*1p36.12*	*KIF17*	*rs2296225*	?	1 × 10^−7^	1.626	[72]
*1p36.13*	*IFFO2*	*rs28530674*	?	3 × 10^−7^	1.826	[72]
*2p22.2*	*PRKD3*	*rs143457389*	A	3 × 10^−16^	1.77	[93]
				2 × 10−6	1.91	
*2p23.1*	*CAPN14*	*rs143457388*	A	3 × 10^−16^	1.77	[93]
		*rs149864795*	A	5 × 10^−10^	2.216	[71]
		*rs77569859*	G	3 × 10^−10^	1.98	[72]
*2q12.1*	*TMEM182*	*rs887992*	C	4 × 10^−10^	0.75	[93]
*3q22.1*	*CPNE4*	*rs554318837*	C	4 × 10^−8^	2.88	[93]
*3q26.32*	*?*	*rs6799767*	?	4 × 10^−7^	1.49	[95]
*4q21.1*	*SHROOM3*	*rs13106227*	?	4 × 10^−6^	1.52	[79]
		*rs1986734*	?	1 × 10^−6^	1.54	
*5q14.2*	*?*	*rs1032757*	T	2 × 10^−6^	1.96	[79]
*5q22.1*	*TSLP*	*rs3806932*	?	3 × 10^−9^	1.85	[79]
		*rs3806933*	G	2 × 10^−8^	1.37	[72]
	*TSLP* *WDR36*	*rs252716*	C	4 × 10^−14^	1.516	[71]
	*WDR36* *RPS3AP21*	*rs1438673*	C	1 × 10^−13^	1.43	[93]
				6 × 10^−22^	0.7	
*5q23.1*	*LINC02214*	*rs2055376*	A	7 × 10^−8^	2.3	[72]
*5q23.2*	*LINC02240*	*rs4240384*	?	2 × 10^−7^	1.4326648	[95]
*5q31.1*	*RAD50*	*rs2106984*	A	4 × 10^−8^	1.26	[93]
*6p11.2*	*GAPDHP15*	*rs9500256*	?	5 × 10^−6^	2.04	[79]
*6p21.33*	*SNHG32* *NEU1*	*rs599707*	?	3 × 10^−9^	1.6920472	[95]
*6p22.3*	*BOLA2P3*	*rs1620996*	T	3 × 10^−8^	0.69	[93]
*7p13*	*URGCP-MRPS24* *URGCP*	*rs188483654*	C	9 × 10^−9^	5.68	[93]
*7p15.1*	*JAZF1*	*rs11495981*	?	9 × 10^−7^	1.308	[95]
*7q22.3*	*LARP1BP2* *CCDC71L*	*rs147307036*	A	1 × 10^−8^	8.04	[93]
*8p23.1*	*XKR6*	*rs2898261*	C	5 × 10^−8^	1.35	[72]
*8q22.2*	*MATN2*	*rs2513845*	T	7 × 10^−9^	4.18	[93]
	*ERICH5*	*rs13278732*	T	6 × 10^−6^	1.31	[79]
*8q24.12*	*SNTB1*	*rs11989782*	A	7 × 10^−6^	1.53	[79]
*9p24.1*	*JAK2*	*rs62541556*	T	4 × 10^−8^	1.61	[93]
*10p11.21*	*CCNY*	*rs191051238*	C	4 × 10^−8^	13.2	[93]
*10p12.31*	*MIR4675*	*rs11819199*	G	3 × 10^−7^	1.62	[72]
*10q21.1*	*PRKG1*	*rs185811602*	T	1 × 10^−8^	6.37	[93]
*10q23.1*	*LINC02650*	*rs2224865*	G	9 × 10^−6^	1.44	[79]
*11p15.4*	*RHOG* *STIM1-AS1*	*rs147702004*	T	1 × 10^−8^	1.95	[93]
*11q13.4*	*SHANK2*	*rs182139615*	T	1 × 10^−9^	6.62	[93]
*11q13.5*	*EMSY*	*rs61894547*	T	4 × 10^−11^	2.439	[71]
			T	4 × 10^−13^	1.92	[93]
			T	5 × 10^−15^	1.79	
	*EMSY* *LINC02757*	*rs2155219*	A	4 × 10^−7^	1.37	[72]
	*CAPN5*	*rs77301713*	?	1 × 10^−7^	2.22	[72]
*11q14.2*	*CCDC81*	*rs118086209*	C	2 × 10^−7^	2.19	[72]
*11q21*	*FAM76B*	*rs1939875*	T	3 × 10^−6^	1.54	[79]
*12q13.3*	*STAT6*	*rs167769*	T	2 × 10^−7^	1.351	[71]
			T	2 × 10^−6^	1.36	[79]
*13q12.13*	*WASF3* *GPR12*	*rs146034499*	A	3 × 10^−9^	5.92	[93]
*14q12*	*LINC02588*	*rs8008716*	G	7 × 10^−8^	1.712	[71]
*15q13.3*	*LINC02352* *KLF13*	*rs8041227*	G	6 × 10^−12^	1.52	[72]
*15q22.2*	*RORA*	*rs2279293*	G	5 × 10^−11^	0.69	[93]
*15q22.33*	*SMAD3*	*rs56062135*	T	4 × 10^−12^	1.29	[93]
*16p13.13*	*CLEC16A*	*rs35099084*	C	3 × 10^−9^	0.71	[93]
			T	2 × 10^−12^	0.72	
		*rs12924112*	?	1 × 10^−7^	1.310616	[95]
*16q24.1*	*MEAK7*	*rs371915*	?	2 × 10^−8^	1.9	[79]
*17q24.3*	*CALM2P1*	*rs6501384*	T	6 × 10^−6^	1.41	[79]
*17q25.3*	*CEP295NL* *TIMP2*	*rs3744790*	?	8 × 10^−7^	1.54	[72]
*18q12.1*	*DSG1*	*rs7236477*	G	7 × 10^−6^	2.22	[79]
*18q12.2*	*INO80C* *GALNT1*	*rs534845465*	A	2 × 10^−8^	5.78	[93]
	*DCC*	*rs9956738*	?	4 × 10^−7^	2.472	[72]
*19q13.11*	*ANKRD27*	*rs3815700*	C	2 × 10^−9^	1.618	[71]
*21q22.3*	*HSF2BP*	*rs17004598*	C	1 × 10^−7^	2.57	[72]
*22q11.21*	*P2RX6*	*rs2075277*	?	9 × 10^−7^	1.544	[72]

## Data Availability

The data presented in this study are available on request from the corresponding author.
